# Adoption of telepharmacy among pharmacists, physicians, and nurses at Hawassa City Public Hospitals, Ethiopia

**DOI:** 10.1371/journal.pdig.0000693

**Published:** 2024-12-11

**Authors:** Jenberu Mekurianew Kelkay, Henok Dessie Wubneh, Henok Molla Beri, Abel Melaku Tefera, Rediet Abebe Molla, Addisu Alem Negatu

**Affiliations:** 1 Department of Health Informatics, School of Public Health, College of Medicine and Health Sciences, Dilla University, Dilla, Ethiopia; 2 School of Medicine, College of Medicine and Health Sciences, University of Gondar, Gondar, Ethiopia; 3 School of Medicine, College of Medicine and Health Sciences, Jimma University, Jimma, Ethiopia; Iran University of Medical Sciences, ISLAMIC REPUBLIC OF IRAN

## Abstract

Pharmaceutical care in the majority of developing countries is hindered by a lack of techniques, limitations in mobility, and a shortage of staff to provide patient care. However, there is no evidence that professionals intend to use telepharmacy in patient care. To fill this gap, this study was designed to examine whether pharmacists, physicians, and nursing professionals intend to use telepharamcy in their care practice.A cross-sectional investigation was carried out from November 29 to December 30, 2023. A study was conducted at all Hawassa public hospitals. A total of 592 Pharmacists, Physicians, and nurses participated. Simple random sampling and proportional allocation were utilized. A structured self-administered questionnaire was used, and a 5% pretest was administered. The data were entered into Epi Data 4.6 and exported to SPSS 26. The AMOS 23 SEM was also used to describe and assess the degree and significance of the relationships between variables.51.4% (304/592) (95% CI, 47.2–55.4) of the participants intended to use telepharmacy. Performance expectancy (β = 0.23, p-value <0.05), social influence (β = 0.295, p-value <0.05), and digital literacy (β = 0.309, p-value <0.001) had positive relationships with the intention to use telepharmacy. Age and gender were also moderators of performance expectancy in telepharmacy.Overall, Pharmacists’, Physicians’, and nurses’ intentions to use telepharamcy were found to be promising for the future. Performance expectancy, social influence, and digital literacy had a significantly positive influence on the intention to use telepharamcy. Digital literacy had a more significant prediction power than others. The results could be useful in terms of designing emerging systems and understanding users’ computer skills.

## Author summary

This study examines the adoption of pharmacists, physicians, and nurses’ professionals to use telepharmacy in patient at Hawassa public hospitals, Ethiopia. In spite of the potential benefits, telepharmacy adoption in low income countries remains limited due to challenges such as flexibility, staff shortages, and lack of skills. Using cross sectional design data were collected from 592 participants through a structured self-administered survey. The results showed that 51.4% of participants intended to use telepharmacy in their practice. Predictors influencing this adoption included performance expectancy, social influence and digital literacy, with digital literacy emerging as a robust predictor. These results recommend promising potential for telepharmacy adoption among healthcare professionals, underling the importance of digital literacy in shaping attitude towards telepharmacy use. The findings can direct the design of telepharmacy systems and inform strategies to enhance technological skills for Pharmacists, Physicians and nurses’ professionals. Healthcare professionals who use telepharmacy in their practice, think about developing incentive programs like continuing medical education credits or awards for reaching telepharmacy-related milestones.

## Introduction

Currently, the delivery of healthcare at a distance has been growing globally for many years [1]. During the past decade, telemedicine has expanded steadily as costs have decreased down and communication technology has improved [2]. Telepharmacy is a subset of telemedicine, which is the delivery of pharmaceutical care through the use of information communication technologies at a remote to patients [3]. Telepharmacy has been used to solve the scarcity of pharmacists and offer pharmaceutical services to those without access to opportunities [3]. Pharmacists are crucial in maintaining pharmaceutical care as they act as a vital link between patients, nurses, and physicians [4].

Despite pharmacists’ important function as healthcare providers, there is an unequal number of these establishments in industrialized nations, with scarcity in both urban and rural areas and at the regional level [5]. Worldwide the distribution of pharmacists according World Health Organization (WHO) was one pharmacist per 10000 population over the period 2006–2012 [6]. The health workforce challenges are migration, lack of supportive policy action, funding, shift to urban areas, and issues of productivity all of which could affect the health care system [7]. The growing demand for pharmacists, nurses, and physicians is promoting the development of telepharmacy as a means of addressing patient care needs [3]. A study in Jordan found that 70.6% of users believe telepharmacy promotes convenient health care service remotely in crises [8]. Findings conducted in different nations, telepharmacy is a rapidly expanding area with evidence strongly supporting its application [9–11].

Pharmaceutical care wants to broaden to the rural area communities to improve patient treatments. As a consequence Telepharmacy services such as remote patient consultation, dispensing drugs, medication orders, medication history review, and medication therapy management come to be common [3]. These services can be delivered by using digital health tools like mobile consultation and software applications [12]. Nevertheless, the advancement of telepharmacy systems, a study in Malaysia indicated that community pharmacist have perceived barriers toward telepharmacy implementation [13]. Also, in a study conducted in China, the consumers have a low level of adoption of health technology [14]. The gap between the intent to use telepharmacy and practitioners was affected by a lack of technological standards, poor internet connections, scepticism between health professionals and patients, and technical problems [3,15]. However, many developed nations are utilizing digital health technologies to improve patient care [16,17].

Even though in Africa health technologies face several challenges policy and regulatory barriers, infrastructural barriers, and organizational and financial barriers [18,19]. A study done in Nigeria’s adoption of telepharmacy was affected by private concerns, regulatory problems, and lack of technological standardization [20]. Similarly, in Ethiopia, the adoption to use telepharmacy among pharmacy students was reported to be low at 48.8% [21].

The consequence of low adoption of telepharmacy technology leads to low-efficiency use of health resources [22], increased care costs, patient waiting times, and limited healthcare access, which leads to low-quality healthcare services [23]. Research in diverse countries showed that performance expectancy, effort expectancy, social influence, and facilitating conditions determine the progress of intention to telepharmacy system among the healthcare workforce [24–26]. Rapid scientific progress and healthcare expenses have directed health work force staffs to uses information technology tools in their work to answer properly to patients [27]. Telepharmacy is a healthcare support method that can address high demand and address challenges due to geographical distances and transportation issues [28]. However, there is inadequate evidence on the adoption of telepharmacy among health professionals in Ethiopia. To fill this information gap, this study was designed to examine whether pharmacists, physicians, and nursing professionals intend to use telepharmacy in their care practice.

Ethiopia’s Federal Minister of Health is considering a national e-health plan to integrate and implement digital health systems in various areas, including pharmaceutical care [29,30]. Telepharmacy offers a significant advantage by providing pharmaceutical care in areas with limited professionals or challenging patient access. The objectives of this study were to determine behavioral intention to use tele pharmacy that introduces an adapted The unified theory of acceptance and use of technology (UTAUT) model and analyze relationships between important predictors of behavioral intention to use tele pharmacy among healthcare professionals.

## The model’s theoretical basis and the research hypothesis

The UTAUT is a widely used model that explains how individuals begin to use new technologies [31]. Compared to the TAM model, which has an average explanatory power of 50% regarding behavior intention to use, the UTAUT has an explanatory value that is 20%–30% greater [31]. The healthcare industry is making active use of studies on end-user acceptance and usage. [32]. Individual difference variables, namely, age [33], and gender are theorized to moderate various UTAUT relationships [34]. But telepharmacy system was not familiar to most people, and due to that experience was excluded as moderator. However, we modified the UTAUT model by adding digital literacy and included important variables that indicate the intention to use telepharmacy. Users with greater IT familiarity are more likely than those with less IT familiarity to accept and stay with new telepharmacy advances [35].

### Factors affecting the intention to use telepharmacy

#### Performance expectancy (PE)

PE is defined as the extent to which consumers will benefit from employing a technology when carrying out specific tasks [34]. A study performed in Italy [27] and Australia [33] revealed that PE was the greatest factor affecting pharmacists’ decision to use telepharmacy. A study conducted in Indonesia [26] found that intention to use cloud-based health services was not influenced by PE. Therefore, this study proposed the following hypothesis:

**H1.** Performance expectancy has a positive influence on intention-use telepharmacy among pharmacists, nurses, and physicians professionals.

## Effort expectancy (EE)

Effort expectancy is the extent to which a person thinks that utilizing a specific digital technology won’t take a lot of work [34]. Studies indicate that EE is the most crucial factor influencing the decision to utilize telepharmacy [27,36]. On the other hand, in Australia, the use of teledentsitery was not influenced by EE [37].

**H2.** Effort expectancy has a positive influence on intention-use telepharmacy among pharmacists, nurses, and physicians professionals.

## Social influence (SI)

Social influence is the extent to which a person believes that important people trust they would use a specific technology [34]. The studies have shown the positive influence of social influence on the intent to use telepharmacy [26,38]. A comparable study in Indonesia indicated that social influence did not influence the adoption to use telehealth [39].

**H3.** Social influence has a positive influence on intention-use telepharmacy among pharmacists, nurses, and physicians professionals.

## Facilitating condition (FC)

Facilitating condition is defined as how customers view the tools and assistance they can use to carry out a task [34]. Various studies have supported the importance of facilitating conditions for the intention to use telepharmacy [26]. A study conducted in Asia found that the relationship between facilitating conditions and the Internet of Things is not significantly influenced [40]. Therefore, we formulated the following hypothesis:

**H4**. Facilitating conditions has a positive influence on intention-use telepharmacy among pharmacists, nurses, and physicians professionals.

## Digital literacy (DL)

This refers to a personality’s ability to gather, assess, and communicate information using various digital media and writing [41–43]. With this model, to test the result of digital literacy on the intent to use telepharmacy technology, the following hypothesis was proposed.

**H5**. Digital literacy has a positive influence on intention-use telepharmacy among pharmacists, nurses, and physicians professionals.

## Methods and materials

### Study design and settings

A cross-sectional design was conducted to assess pharmacists’, nurses’, and physicians’ intentions to use telepharmacy in the Hawassa City public hospitals, in Ethiopia. Hawassa is the capital city of Sidama Regional State. Based on the Hawassa City Administration Health Department 2023 in the city, there are 11 health centers and 4 public hospitals including Hawassa University Comprehensive Specialized Hospital, Adare General Hospital, Motite Furra Primary Hospital, and Tula Primary Hospital in Hawassa City. This study was conducted from November 29 to December 30, 2023, at all public hospitals in Hawassa City, Sidama, Ethiopia. Currently, approximately 1015 nurses, pharmacists, and physicians have worked in those hospitals.

### Populations

All pharmacists, nurses, and physicians who were working at public hospitals in Hawassa City were included in the sample. The study populations were all pharmacists, nurses, and physicians who had worked at public hospitals in Hawassa City during the data collection period and were contacted via in-person recruitment, excluding those with less than 6 months of experience.

### Sample size determination

**Fig 1:**
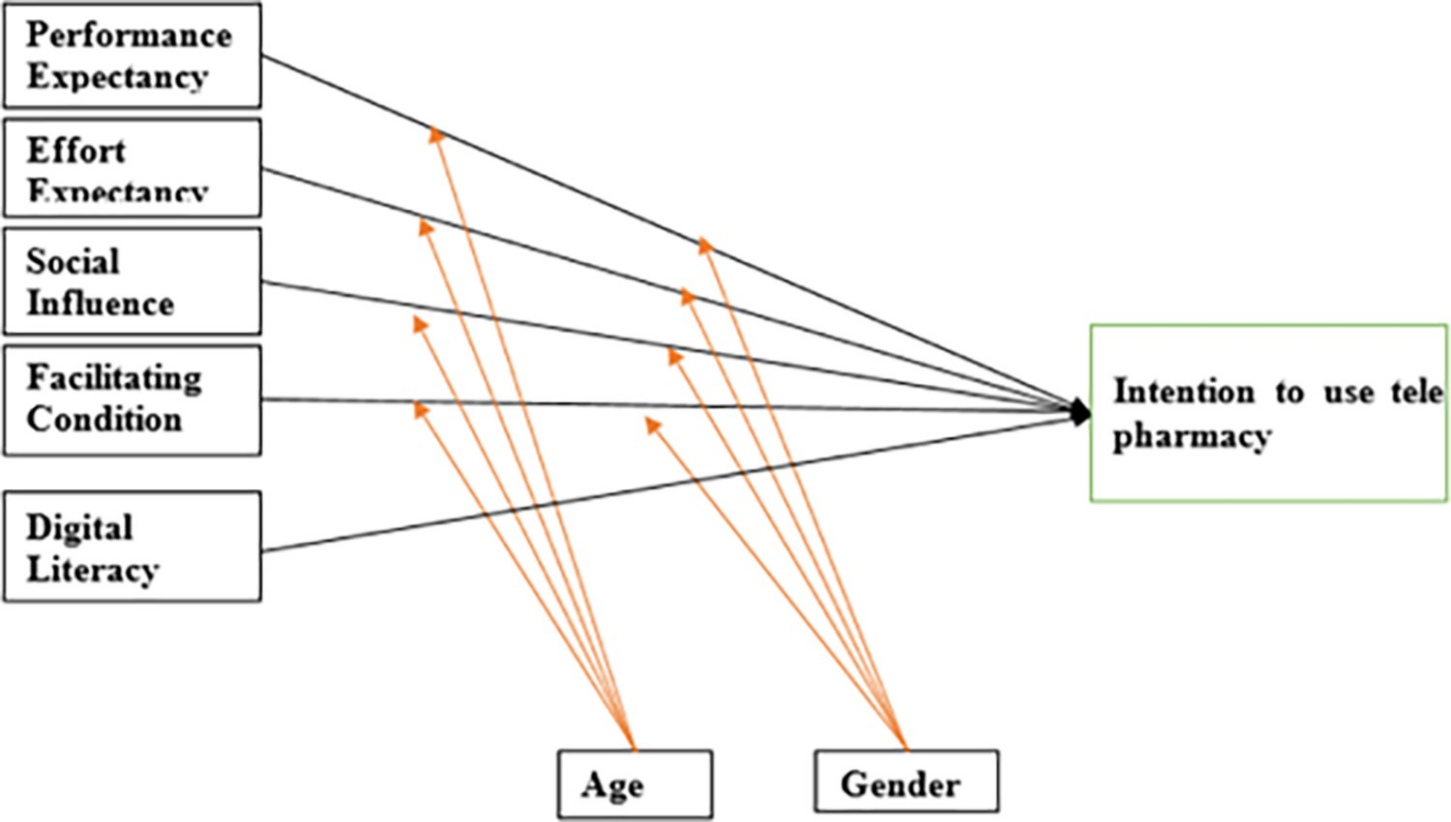
Conceptual framework for intention to use telepharmacy among pharmacists, nurses and physicians.

The proposed model has 85 parameters (57 free parameters and 28 fixed parameters), as computed using AMOS version 23. The free parameters include 22 error terms, 15 free factor loadings, 5 variances of an independent variable, 10 covariances, and 7 regression coefficients. The quantity of free parameters in the theoretical model was used to calculate the minimal sample size; it has been suggested that the proportion of responders to free variables to be calculated is 1:10 [44]. Hence, taking into account the 57 parameters that would be calculated using the proposed model using Fig 1 and getting participants to a free parameter ratio of one to ten, 570 samples are required as a minimum. The final sample size, which accounts for a 10% nonresponse rate, was calculated to be 627.

### Sampling Procedure

Study respondents were selected from all public hospitals in Hawassa city. Simple random sampling was used, with proportional allocation for each health facility. Finally, the open random program V.3 randomly selected each participant from each public hospital.

### Variables of the study

Intention to use telepharmacy is the dependent variable and the independent variables are performance expectancy, effort expectancy, social influence, facilitating condition, digital literacy and pharmacists, nurses and physicians ’ sociodemographic characteristics: sex, age, marital status, educational status, and monthly income.

### Operational definition

Intention to use telepharmacy: The degree to which a nurse has made deliberate choices to participate in or avoid a particular future action when utilizing telepharmacy [45].

Intention to use: means in this study refers to the likelihood of pharmacists, nurses, and physicians intended or not to use telepharmacy if they were offered the construct has 3 items, and each was evaluated by a 5-point Likert scale (1) strongly disagree, (2) disagree, (3) neutral, (4) agree, (5) strongly agree response the median was used as a cutoff point [26]. A nurse who scores “median” or “above” on the intention to use a construct was considered to intend to use telepharmacy; otherwise, it was considered unintended.

### Data collection tools

We employed a standard questionnaire in this study, which was modified from earlier research and Venkatesh’s original work. [34,46–48]. Cronbach alpha and composite reliability were used to evaluate the concept validity of CFA and reliability. The tool was written in the English version because it is the common language in Hawassa city. The questionnaire consists of gender, age, marital status, educational level, and monthly income are the user demographics and symbolize the constructs included in the UTAUT represented by the 21 positive statements. There are a total of 26 items in the questionnaire, and the constructs were measured using a five-point Likert scale, with 1 denoting strongly disagree and 5 denoting strongly agree [34,49].

### Data collection procedures

A self-administered questionnaire was administered under MPH supervision, with data collected by BSc/HI and BSc/pharmacists and nurses professionals. To help the respondents understand the relevance of the survey questions, supervisors and data collectors were trained on research aims and the use of telepharmacy for prescription services. As a result, the respondents either accepted to participate in the study or refused to do so. Those who did not give their consent were thanked for their participation.

### Data quality assurance

To ensure the quality of the data, a pretest was administered to 5% of Dilla referral hospital pharmacists, nurses, and physicians, ensuring data quality. Cronbach’s alpha values were above 0.7, achieving internal consistency, with slight adjustments to questionnaire wording and comprehensibility. Four data collectors and two supervisors were trained on study objectives, procedures, confidentiality, and respondents’ rights. The data collection process was thoroughly monitored and its completeness was verified post-collection.

### Data processing and analysis

The data was coded using Epi-data version 4.6, analyzed using SPSS version 26 for descriptive analysis, and assessed using AMOS version 26 for structural and measurement model assessment. No missing values were found. A frequency table displayed findings. The assumption was checked and the maximum likelihood estimate approach was used [50]. The structural equation model assumed multiple measurements for each latent variable, which was met with three or more observed variables. The Variance Inflation Factor (VIF<10 and tolerance>0.1) was used to evaluate the multicollinearity [51]. The study used kurtosis and skewness scores to assess multivariate normality but found it unsatisfactory, requiring bootstrapping to manage multivariate nonnormality [52].

The measurement model was assessed using confirmatory factor analysis, Cronbach’s alpha, and composite reliability, with an internal consistency cut-off point above 0.7[53]. Convergent validity was assessed using factor loading and the average variance extracted [51]. An AVE score of more than 0.50 in the measurement model assessment [54] would be used to begin convergent validity [55]. Discriminant validity evaluates the distinctiveness of an assumption by its items, using the square root of the AVE, which should be greater than inter-construct correlations [56].

The measurement model utilized the model’s goodness of fit [43]. The model’s goodness-of-fit was measured using the following measurements such as chi-square to a degree of freedom ratio <3, a standardized root mean square residual< 0.08, a root mean square error of approximation < 0.08, a comparative fit index > 0.90, a goodness-of-fit index > 0.90, an adjusted goodness-of-fit index > 0.85, a normalized fit index > 0.90 [57]. The model modification was modified to enhance its fitness by removing factors with a factor loading value < 0.5 or covarying error terms [58]. Standardized path coefficient, and significance level to test hypotheses and determine associations between latent variables, with standardized regression weights indicating the strength of association [59], and a p-value < 0.05 indicating the level of significance. The square multiple correlations were used to report the proportion of variance in the endogenous latent variables explained by the exogenous variables [60].

### Ethics approval and consent to participate

The Institutional Ethical Review Board of Dilla University College of Health and Medicine Sciences provided ethical approval (IRB 0103/20-16), which the study was performed according to the Declaration of Helsinki. Criteria. Each participant in the study provided his or her written consent after being informed of the study’s goal. Furthermore, all the data collectors and investigators strictly adhered to the law’s requirements for data privacy and confidentiality. Only the study was conducted using the data that were retrieved. As a result, the data collection tool did not contain participants’ names or any other personal information about them. Also, this study was not an experimental.

## Results

627 respondents were planned to be involved in the study. Among these, 94.4% had a response and more than half (57.3%) of the respondents were male. The majority of the respondents were aged between 20 and 29 years (Table 1).

**Table 1:** Sociodemographic characteristics of pharmacists, physicians and nurses in Hawassa city public hospitals, Ethiopia, 2023 (n = 592).

Sociodemographic Characteristics	Category	Frequency (n)	Percentage (%)
Gender	Male	339	57.3
	Female	253	42.7
Age	20–29	268	46.6
	30–39	262	44.3
	40–49	42	7.1
Marital status	Single	261	42.4
	Married	277	46.8
	Other*	64	10.8
Educational status	Diploma	55	9.3
	Bachelor degree	495	83.6
	Master’s and above	42	7.1
Monthly salary	≤ 5000	41	6.9
	5001–10000	475	80.2
	> 10000	76	12.8

Other* (widowed, separated)

### Intention to use telepharmacy

Based on the results of this study, 304 (54.4%) pharmacists, nurses, and physicians (95.0% CI: [47.2–55.4]) were intended to use telepharmacy system. Three-item questions with five Likert ratings were utilized for assessing behavioral intention to use telepharmacy. The intention of using telepharmacy had a median score of 10 (interquartile range (IQR): 7–12), and the maximum and minimum scores were 15 and 3, respectively.

### Measurement model assessment

**Fig 2:**
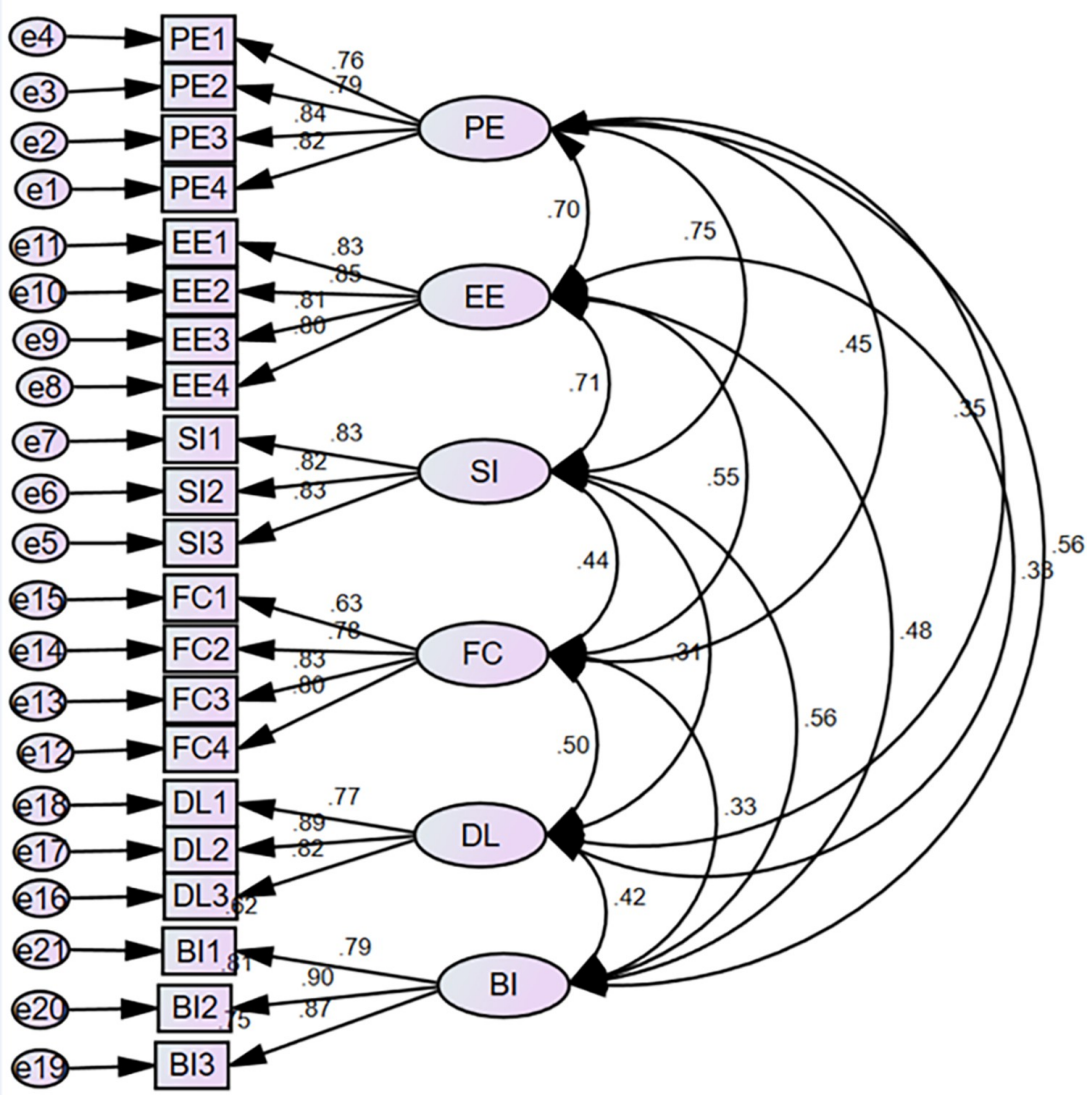
Confirmatory factor analysis of the intention to use telepharmacy among healthcare pharmacists, nurses and physicians in Hawassa city public hospitals, Ethiopia, 2023.

The composite reliability and Cronbach’s alpha values in this study were both higher than 0.8. All the factor loading values ranged from 0.63 to 0.89, and the AVE ranged from 0.582 to 0.730 (Fig 2). Thus, the measurement model’s construct reliability and convergent validity were achieved. The test of sampling adequacy **Kaiser–Meyer–Olkin** of all the constructs was also greater than 0.7, as shown in Table 2.

**Table 2:** Factor loading, composite reliability, average variance extracted, and Cronbach’s alpha of the construct of the proposed model.

Construct	Indicators/Items	Factor loadings	Composite Reliability (CR)	Cronbach Alpha	Average Variance Extracted (AVE)	Kaiser–Meyer–Olkin (KMO)
PE	PE1	0.76	0.880	0.879	0.647	0.812
	PE2	0.79				
	PE3	0.84				
	PE4	0.82				
EE	EE1	0.83	0.866	0.893	0.683	0.834
	EE2	0.85				
	EE3	0.81				
	EE4	0.80				
SI	SI1	0.83	0.894	0.866	0.678	0.739
	SI2	0.82				
	SI3	0.83				
FC	FC1	0.63	0.846	0.842	0.582	0.802
	FC2	0.78				
	FC3	0.83				
	FC4	0.80				
DL	DL1	0.77	0.866	0.864	0.684	0.722
	DL2	0.89				
	DL3	0.82				
BI	BI1	0.79	0.890	0.877	0.730	0.733
	BI2	0.80				
	BI3	0.87				

### Discriminant validity

The study’s results indicated that the measurement model’s discriminant validity is achieved, as the square root of the AVE value exceeded the interconstruct correlations value (Table 3). The square root of the average variance extracted is indicated by the bolded value in the table.

**Table 3:** Results of discriminant validity of the proposed model.

Construct	PE	SI	EE	FC	DL	BI
**PE**	**0.804**					
**SI**	0.753	**0.826**				
**EE**	0.689	0.710	**0.824**			
**FC**	0.451	0.439	0.551	**0.763**		
**DL**	0.353	0.306	0.329	0.495	**0.827**	
**BI**	0.555	0.561	0.477	0.327	0.423	**0.854**

### Measurement indices of the goodness of fit of the model

In this result, all of the measurement model fit indices’ obtained values were Chia-square/degree of freedom = 2.711, the goodness-of-fit index = 0.930, Normed fit index = 0.941, Adjusted goodness-of-fit index = 0.906, Comparative fit index = 0.962, Standardized room mean squared residual = 0.023 and Root mean square errors of approximation = 0.054. Based on the goodness of fit model’s value met the requirements.

### Structural Model Assessment

**Fig 3:**
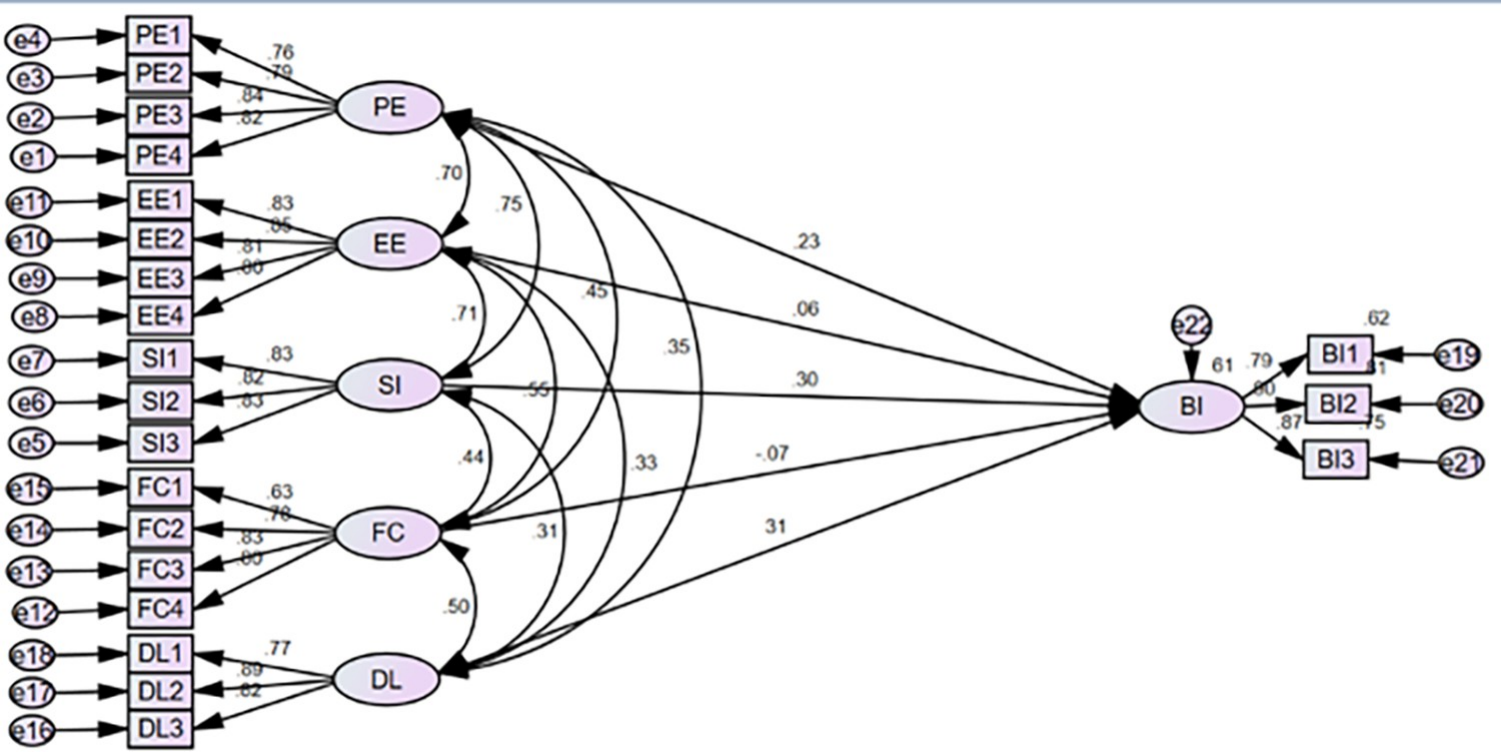
SEM for predictors of intention to telepharmacy among pharmacists, nurses and physicians in Hawassa city public hospitals, Ethiopia, 2023.

In this finding, the proposed model explained 61% (**R**^**2**^ **= 0.61)** of the variance in the intention to use telepharmacy (Fig 3). Among the six proposed hypotheses of the direct relationship, three were supported by the collected data. The results of the study showed that DL (β = 0.309, 95% CI: [0.186, 0.366], p value<0.001) had the top significant effect on participants’ intention to employ telepharmacy, which exceeded the influence of the other variables. In the same way, the desire to utilize telepharmacy was significantly influenced by PE (β = 0.0.230, 95% CI: [0.027, 0.427], p value<0.05) and SI (β = 0.295, 95%yhj CI: [0.126, 0.426], P value<0.05 (Table 4).

**Table 4:** SEM analysis of predictors of intention to use telepharmacy among pharmacists, nurses and physicians at public hospitals in the Hawassa city, Ethiopia, 2023.

Hypothesis	Path	Path Coefficient	S.E	C.R	P value	95% confidence interval		Result
						Lower	Upper	
H1	BI ← PE	0.230	0.069	3.218	0.025	0.027	0.427	supported
H2	BI ← EE	0.058	0.067	0.863	0.500	-0.122	0.251	Not supported
H3	BI ← SI	0.295	0.072	4.005	0.002**	0.126	0.426	supported
H4	BI ← FC	-0.071	0.053	-1.237	0.246	-0.186	0.051	Not Supported
H5	BI ← DL	0.309	0.042	5.681	***	0.168	0.366	Supported

^**^p-value <0.01

^***^ p-value <0.001 S.E: standard error, C.R: critical ratio.

### Testing potential moderation

In this section, an investigation was conducted into the potential moderating effects of age and sex on the relationship between each independent variable with the dependent variable.

### Moderating effect of gender

The positive influence of performance expectancy on the intention to use telepharmacy was positively moderated by gender and was significantly stronger for male participants (β = 0.088, p value<0.05) than for women participants (β = -0.008, p value = 0.949) (Table 5).

**Table 5:** Moderating effects of gender on the intention to use telepharmacy among pharmacists, nurses and physicians at public hospitals in the Hawassa city, Ethiopia, 2023.

Hypothesis	Moderator	Path coefficients	P value	Model test (unconstrained and constrained model)		Result
	Gender			Δ X^2^	P Value	
BI← PE	Male	0.088	0.003**	5.555	0.001**	Supported
	Female	-0.008	0.949			
BI ← EE	Male	0.060	0.364	0.446	0.506	Not supported
	Female	-0.008	0.949			
BI← SI	Male	0.294	0.001**	0.001	0.978	Not supported
	Female	0.290	0.014			
BI ← FC	Male	-0.044	0.509	0.066	0.797	Not supported
	Female	-0.073	0.427			
BI ← DL	Male	0.282	***	1.511	0.219	Not supported
	Female	0.177	0.004**			

^**^ Significance at p-value <0.01

*** Significance at p-value <0.001.

### The moderating effect of age

The positive influence of predictors on the intention to use telepharmacy was not positively moderated by age.

## Discussion

The objective of this study was to investigate the intent to use telepharmacy and its predictors among pharmacists, nurses, and physicians in public hospitals. According to findings, 304 pharmacists, nurses, and physicians were likely to intend to use telepharmacy (51.4%). A study showed that performance expectancy, perceived enjoyment, and digital literacy have a positive relationship with intention to use telepharmacy. A study shows half of pharmacists, nurses, and physicians plan to use telepharmacy services for patient healthcare, indicating a potential high-impact digital health solution that can be scaled up over the next decade [30]. Moreover, telepharmacy system is still in its infancy stage of development in Ethiopia [61]; considering this, our findings on the intention to use telepharmacy among pharmacists, nurses, and physicians are promising for the future. A study conducted in Ethiopia revealed a score of 39.8% [43], which is lower than that in our study. The discrepancy may be due to the majority of study participants’ lack of familiarity with technological devices. Another reason for this discrepancy could be the small sample size (n = 423).

Performance expectancy directly influenced pharmacists, nurses, and physicians ’ intention to use telepharmacy (p value<0.05). This shows that pharmacists, nurses, and physicians are motivated to increase productivity by improving pharmaceutical care and making it easier to prescribe challenging cases, and they are interested in how telepharmacy services could assist their everyday work. These findings are consistent with those of several studies conducted in Italy [26], Australia [33], and a study done in Chile adoption of eHealth among users [62]. This result suggested that when telepharmacy technology is thought to be beneficial to their practice and could improve their job performance and productivity, pharmacists, nurses, and physicians are more likely to intend to use it. In contrast, these findings contradict those of a study on telemedicine during the COVID-19 epidemic in Ethiopia (p value<0.05) [63]. This discrepancy could be explained by the use of internet-based questionnaires for the investigation.

The study found that social influence directly influences pharmacists’, nurses’ and physicians ’ intention to use telepharmacy, with individuals with significant user impact encouraging this technology use (P < 0.05). These results are in line with earlier research carried out in several nations, such as Ethiopia [43], Iran [64], Austria [36], and Italy [27]. A possible reason might be that hospital administration, patients, and medical professionals are pressuring healthcare providers to adopt a new system [65]. However, in an Indonesian study, SI did not influence the participants’ desire to use healthcare technology [39]. The discrepancy might be due to the small sample size, which could have affected the findings.

This finding furthermore revealed that there was a strong association between DL and intention to use telepharmacy (p value<0.001), which had an influence on intention that was larger than that of the other factors. The finding suggested that pharmacists, nurses, and physicians were likely to plan to use telepharmacy systems if they were familiar with various digital technologies and needed to use them for work. This finding was consistent with the findings of studies performed in Ethiopia [43] and Saudi Arabia [66]. Moreover, pharmacists, nurses, and physicians can use telepharmacy technology to improve patient outcomes if they have the necessary skills to access, comprehend, evaluate, and use pharmaceutical care on the Internet.

According to the SEM analysis, the association between performance expectancy and intention to use telepharmacy technology was positively moderated by sex (p<0.01). This finding showed that among pharmacists, nurses, and physicians who intended to use telepharmacy services, there was a significant difference in performance expectations between male and female pharmacists, nurses, and physicians. It was mentioned that females typically find it more difficult to utilize new information systems and perceive them as being less helpful for their tasks [67,68]. The reason for this difference might be due to men’s connections with digital health knowledge; males are more mindful of internet healthcare than females [69].

### Implication of the study

The study suggests that healthcare decision-makers, managers, and facilities can improve telepharmacy technology adoption and utilization by ensuring quick service completion and encouraging knowledge sharing among users. This could also improve technology usage, aiding in the decision-making process for health information system development and system design. The decision-makers, pharmacists, and healthcare professionals could believe that telepharmacy will enable them to give patients faster, better treatment, they are more inclined to embrace it. Telepharmacy can shorten patient wait times by facilitating faster service completion, which improves patient satisfaction and health outcomes. Health care professionals can readily adjust to any updates or modifications in the telepharmacy system if they receive ongoing training and user assistance, which can further reduce the perceived effort involved in using the technology. The adoption of telepharmacy systems in healthcare organizations is greatly influenced by the support of peers, managers, or other reputable medical professionals. Influential people who actively support telepharmacy instill a sense of social responsibility and normative pressure on other staff members to do the same. Since people are more inclined to adopt a system they believe to be effectively used by reliable colleagues, this dynamic can greatly accelerate the dissemination of telepharmacy technology. Easy-to-use, visually appealing, and responsive telepharmacy systems can greatly improve user experience by making jobs more pleasurable in addition to being more productive. Users are more likely to interact with a system beyond completing tasks when it feels current responsive, and easy to use for a good emotional response. People that enjoy using technology are more likely to keep using it, therefore this satisfaction can result in sustained utilization.

## Conclusion and reccomendations

Overall, pharmacists, nurses, and physicians’ intentions to use telepharmacy systems were found to be promising for the future. PE, SI, and DL had a significantly positive influence on the intention to use telepharmacy. Gender significantly influenced the association between PE and intention to use telepharmacy among pharmacists, nurses, and physicians. Digital literacy had a more significant prediction power than others. The results could be useful in terms of designing emerging systems and understanding users’ digital skills. For healthcare professionals who use telepharmacy in their practice, think about developing incentive programs like continuing medical education credits or awards for reaching telepharmacy-related milestones. Understanding the application of telepharmacy can encourage professionals to interact with the platform more thoroughly. Conduct longitudinal research to observe change in perceptions of telepharmacy over time. This would shed light on how social influence, subjective enjoyment, and performance expectancy change as technology and familiarity increase.

## Supporting information

S1 Dataset Data set of pharmacists’, nurses’ and physicians’ adoption of telepharmacy in Hawassa city public hospitals, Ethiopia. (SAV)
